# Peritumoral Tertiary Lymphoid Structures Correlate With Protective Immunity and Improved Prognosis in Patients With Hepatocellular Carcinoma

**DOI:** 10.3389/fimmu.2021.648812

**Published:** 2021-05-26

**Authors:** Hui Li, Hailing Liu, Hongyuan Fu, Jiaxin Li, Lin Xu, Genshu Wang, Hong Wu

**Affiliations:** ^1^ Department of Liver Surgery, Liver Transplantation Division, West China Hospital, Sichuan University, Chengdu, China; ^2^ Laboratory of Liver Surgery, West China Hospital, Sichuan University, Chengdu, China; ^3^ Department of Hepatobiliary Pancreatic Tumor Center, Chongqing University Cancer Hospital, Chongqing, China; ^4^ Department of Hepatobiliary Surgery, Affiliated Tumor Hospital of Guangxi Medical University, Nanning, China; ^5^ Department of Hepatic Surgery and Liver Transplantation Center, the Third Affiliated Hospital of Sun Yat-sen University, Guangzhou, China

**Keywords:** tertiary lymphoid structure, tumor immunology, immune infiltration, active immune reaction, hepatocellular carcinoma

## Abstract

The existence of intratumoral tertiary lymphoid structure (iTLS) has been reported to correlative with favorable clinical outcomes for patients with hepatocellular carcinoma (HCC). However, little is known about the role of peritumoral TLS (pTLS). This study aimed to investigate the prognostic role of pTLS either alone or jointly with iTLS and the potential association with local immune response in HCC. The formation and cellular composition of TLS was evaluated by hematoxylin & eosin and immunohistochemistry. Evaluation of tumor-infiltrating immune cells and formation of germinal center (GC) inside TLS was performed by immunohistochemistry. The gene expression profiles were analyzed by real-time PCR. In a total of 360 patients from two independent cohorts, the pTLS was identified in most, whereas iTLS could be observed in only approximately 30% of HCC specimens. Patients with high pTLS densities were associated with improved outcomes, those present with characteristic morphology of GC, particularly, showing an even better prognosis. The combination of pTLS and iTLS allowed the identification of patients with best prognosis. Tumors with high pTLS density showed significantly increased expression of Th1-, Th17- and immune suppression-related genes, as well as significantly higher infiltration of CD3+, CD8+ and CD20+ cells and lower infiltration of FOXP3+, CD68+ and PD1+ cells. Conclusively, we provide evidence that pTLS is associated with intratumoral immune infiltration, highlighting the dynamic interplay between pTLS and immune cells recruitment. High pTLS density links to a tumor microenvironment with an active immune reaction and improved patient survival and represents a promising prognostic biomarker for HCC.

## Background

Hepatocellular carcinoma (HCC) is the sixth most common human cancer, causing for the fourth most cancer-related mortality globally (1). The prognosis of HCC patients remains poor due to rapid progression and frequent incidence of recurrence (2). It is necessary to elucidate the histological features more profoundly to identify effective therapeutic strategies against HCC progression. Given the established significance of host immune system and tumor microenvironment regarding tumor initiation and development, mounting studies have investigated the influence of tumor-infiltrating lymphocytes (TIL) on patients’ clinical outcomes in various malignancies (3, 4). Apart from TIL, recent evidence demonstrated that the spatial organization of immune cells also played a major role in determining tumor invasion and metastasis (5, 6).

Tertiary lymphoid structures (TLSs) are ectopic lymphoid organs that exhibit similar morphological, cellular, and molecular properties to secondary lymphoid organs, especially lymph nodes (7). The formation of TLS was observed in autoimmune diseases, allograft rejection and non-lymphoid tissues at sites of chronic inflammation including tumors (8, 9). Tumor-associated TLS, characterized by B cell lymphoid follicles with or without germinal centers, T cell-rich zones, and specialized vessels known as high endothelial venules (HEVs), provide necessary specialized vasculature and chemoattractant, allowing for immune cells infiltration (10, 11). A high density of TLS-associated mature dendritic cell (DC) correlated closely to a strong infiltration of T cells as well as expression of genes related to T-cell activation, T-helper 1 (Th1) phenotype, and cytotoxic orientation (12). In addition, the HEVs could specially express peripheral node addressin and L-selectin ligands (11, 13). CXCL13 expressed by follicular DCs facilitates B cells into tumor and formation of germinal center, while CCL19 and CCL21 are crucial for recruitment of T cells and DCs, favoring lymphoid neogenesis (14, 15). Accumulating evidence have shown that the presence of TLS, whether in tumor core or peritumoral areas, correlated with favorable clinical outcomes in various cancers, including breast cancer, colorectal cancer and lung cancer, and tumor-infiltrating immune cells (5, 16, 17). However, dual role of TLS has been reported in HCC. Finkin et al. demonstrated that TLSs located in the non-tumoral liver tissue served as niches for malignant hepatocyte progenitors and correlated with an elevated risk of late HCC recurrence (18). In contrast to the findings obtained in the non-tumor liver tissues, intratumoral TLSs (iTLS) were predictive of a decreased risk of early relapse (19). We also investigated the presence of iTLS in HCC tissues and their correlation with clinical outcomes, and found the presence of iTLS correlated with decreased risk of early tumor relapse as well as facilitating immune cells infiltration (20).

As mentioned above, studies in HCC mainly focused on TLS intratumorally, thus, to a certain extent, neglecting their role when they located peritumorally (adjacent to the infiltrative tumor border). In this study, we investigated the presence and density of TLS in HCC intratumoral or peritumoral tissues and their association with tumor-infiltrating immune cells and cytokine milieu. High pTLS density was significantly correlated with active immune reaction and favorable patient survival. Additionally, pTLS facilitated an active immune reaction and various immune cells infiltration.

## Material and Methods

### Patient Cohorts and Samples

Samples were collected from patients who underwent initial surgical resection for HCC in West China Hospital between 2009 and 2013. Exclusion criteria were as follows: preoperative anti-cancer therapies, such as radiofrequency ablation, transarterial chemoembolization and chemotherapy; mixed tumor histology; extrahepatic metastasis at the time of diagnosis. Patients passed the exclusion criteria were selected for analysis and reviewed for their clinicopathological data (training cohort, n=240). The baseline characteristics are summarized in **Table 1**. Patients were followed-up in outpatient clinic per 3 months during the first postoperative year and 6-12 months thereafter, until December 2018. Besides, we contact those who determined not to go back to the hospital through telephone follow-up survey. The median follow-up period was 60.3 (2.4-111.7) months. Overall survival (OS) was calculated from the date of liver resection to death or, in those alive, to the date of last follow-up. Recurrence-free survival (RFS) was from the date of surgical resection to diagnosis of recurrence or last follow-up.

**Table 1:** Baseline characteristics of 240 HCC patients and univariate analysis of factors possibly affecting the survival.

Variables	Number (%)	OS (P value)	RFS (P value)
Age (median, 50)		0.091	0.227
<50	117 (48.8)		
≥50	123 (51.2)		
Gender		0.605	0.179
Male	202 (84.2)		
Female	38 (15.8)		
HBsAg		0.010	0.052
Yes	211 (87.9)		
No	29 (12.1)		
Cirrhosis		0.804	0.465
Yes	150 (62.5)		
No	90 (37.5)		
Portal hypertension		0.616	0.506
Yes	36 (15)		
No	204 (85)		
AFP, ng/dL		0.257	0.002
<400	139 (57.9)		
≥400	101 (42.1)		
Tumor size, cm		0.003	0.003
<5	104 (43.3)		
≥5	136 (56.7)		
Tumor number		0.031	0.014
Single	193 (80.4)		
Multiple	47 (19.6)		
Differentiation		0.479	0.416
Well/Moderate	148 (61.7)		
Poor	92 (38.3)		
Macrovascular invasion		<0.001	<0.001
Yes	12 (5)		
No	228 (95)		
Microvascular invasion		<0.001	<0.001
Yes	81 (33.8)		
No	159 (66.2)		
BCLC stages		0.002	0.004
0-A	185 (77.1)		
B-C	55 (22.9)		
TNM stages		0.127	0.127
I-II	178 (74.2)		
III	62 (25.8)		
pTLS		<0.001	<0.001
Low	89 (37.1)		
High	151 (62.9)		
iTLS		0.121	0.009
Present	76 (31.7)		
Absent	164 (68.3)		
GC		<0.001	<0.001
Present	106 (44.2)		
Absent	134 (55.8)		

AFP, alpha-fetoprotein; BCLC, Barcelona Clinic Liver Cancer; TNM, tumor-nodes-metastasis; pTLS, peritumoral tertiary lymphoid structures; iTLS, intratumoral tertiary lymphoid structures; OS, overall survival; RFS, recurrence-free survival; HCC, hepatocellular carcinoma; GC, germinal center.

For the validation cohort, samples from HCC patients underwent initial hepatic resection at the Third Affiliated Hospital of Sun Yat-sen University between 2009 and 2012 were collected (validation cohort). Detailed information on clinicopathological features is provided in **Supplementary Table 1**. Patients were followed-up until February 2019. The median follow-up period was 62.9 (3.1-113.7) months.

This work was performed in accordance with the guidelines of the 1975 Declaration of Helsinki, and approved by the Institutional Review Board of West China Hospital of Sichuan University and the Third Affiliated Hospital of Sun Yat-sen University.

### Pathological Examination

Pathological examinations were conducted according to the World Health Organization (WHO) classification by two pathologists who were blinded for patients’ clinical outcomes. Tumor stages were evaluated using the tumor-node-metastasis staging system (TNM, 8th edition) and the Barcelona Clinic Liver Cancer (BCLC) staging system. The maximum cut surface of surgically resected tumor block, containing intratumoral area and peritumoral parenchyma, was used for histological evaluation. The presence and location of TLS was assessed in five serial hematoxylin and eosin (H&E)-stained sections. The evaluation of TLS was performed by a pathologist. Given that several cell types, such as B cell lymphoid follicles, T cell zones and mature DCs, have been frequently used as surrogates for qualification of TLS, the presence of TLS in our sections were confirmed by immunochemical staining of CD3, CD20, CD21, CD23 and DC-LAMP. The mean number of TLS was recorded and used for analysis. The density of TLS was calculated as number/mm^2^ in intratumoral (within tumor tissue) and peritumoral (5 mm from the infiltrative tumor border) regions. A patient was considered as intratumoral TLS (iTLS) positive if at least one TLS was observed (18, 21). We also evaluated the presence of germinal center (GC) in HCC tissues by staining with CD21 and CD23 in consecutive sections. As previously described, tumor was considered as GC-positive if at least one TLS (pTLS or iTLS) exhibited the characteristic morphology of proliferating centroblasts (9). Peritumoral TLS (pTLS) was stratified into two groups according to the density. The included HCC tissues were classified into four categories based on the frequency of iTLS and pTLS: iTLS absent and low pTLS density; iTLS absent and high pTLS density; iTLS present and low pTLS density; iTLS present and high pTLS density.

### Immunochemistry

Formalin-fixed, paraffin-embedded (FFPE) tumor blocks were used for preparation of 4-μm thick serial sections. Immunochemistry and quantitation of tumor-infiltrating immune cells was performed as previously described (22). Briefly, for each section, three areas with highest infiltration density were used for evaluation of representative immune infiltrating cell. At least five fields (20× high-power) per area were evaluated for immune markers. The number of stained cells per 20× high-power field was counted and converted to cell density (cell number/mm^2^). The quantification of tumor-infiltrating immune cells was performed by the same pathologist. Slides were stained for CD3 (pan T cell), CD8+ T cell, FOXP3 (regulatory T cell), CD20 (B cell), CD68 (macrophage) and CD57. PD1 and PDL1 were also stained in intratumoral immune cells. The details of primary antibodies for immunochemistry and staining conditions are mentioned in **Supplementary Table 2**.

### Gene Expression Analysis

Total RNA was extracted from frozen HCC tissues by using Cell Total RNA Isolation Kit (Foregene, Chengdu, China) in accordance with the manufacturer’s instructions. The first-strand complementary DNA was synthesized using HiScript II Reverse Transcriptase (Vazyme, Nanjing, China). ChamQTM SYBR@ qPCR Master Mix (Vazyme Biotech, Nanjing, China) was utilized for real-time PCR. All the reactions were performed in triplicate and the relative expression levels were normalized by the expression of U6. Primers used in this study were summarized in **Supplementary Table 3**.

### Statistical Analysis

All the statistical analyses were performed by applying SPSS software (version 23.0, SPSS Inc., Chicago, IL, USA), GraphPad Prism software (version 8.0, La Jolla, CA, USA) and MedCalc software (version 15.2.2). The optimal threshold of pTLS density was identified by application of the receiver operating characteristics (ROC) curve. The 5-year survival status was set as the discriminant. In addition, a minimal p-value approach was used to test the determined cut-off. Mann-Whitney U-test was used to investigate continuous variables with unequal variance. The Spearman correlation or Person χ2 test was applied to examine correlations. Multiple hypothesis test was assessed by using Monte Carlo method. The survival curves were plotted using Kaplan-Meier method and tested by log-rank test. Univariate analysis was utilized for evaluation of prognostic factors of OS and RFS. Those found to be significant in univariate analysis were subjected to multivariate regression models (enter method). A two-tailed P < 0.05 was considered statistically significant.

## Results

### TLS in HCC Tissue

**Figure 1:**
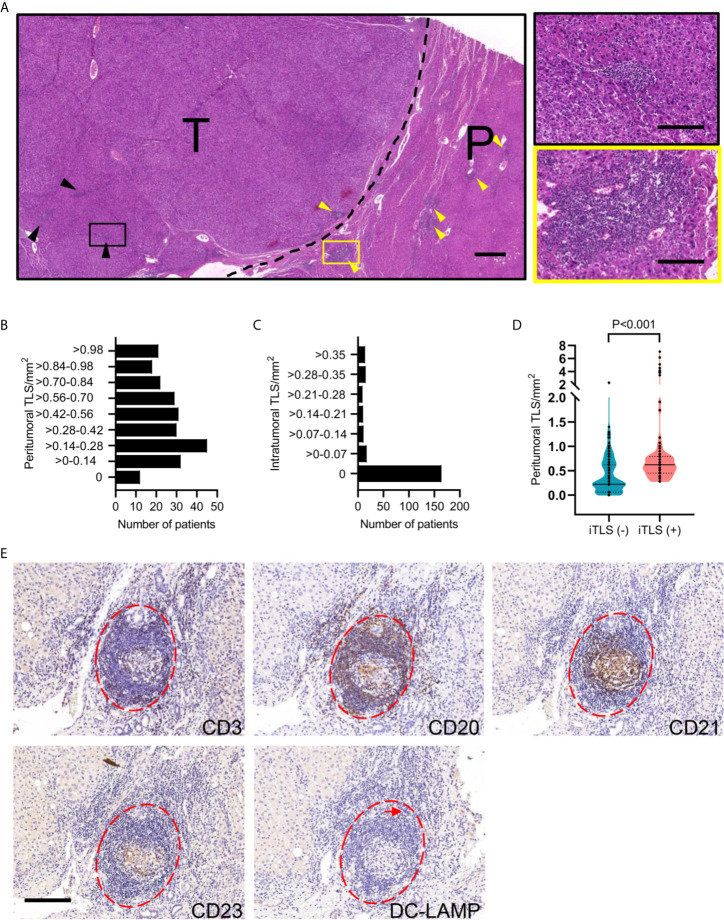
Characterization of HCC-associated TLS. **(A)** Representative images of H&E-stained HCC tissues showing iTLS (black arrowheads and right zoomed-in segment) in tumor tissue and pTLS (yellow arrowheads and right zoomed-in segment) surrounding the HCC tissues. The black dotted line represents the invasive margin of HCC. Scale bar, left 500 μm; right, 50 μm. **(B, C)** The density of TLS was determined in peritumoral and intratumoral regions in 240 patients with HCC. **(D)** The density of pTLS was compared between HCC patients present or absent with iTLS. Groups were statistically compared using Mann–Whitney U-test. **(E)** Representative images showing components and structure of TLS using consecutive sections. Lymphoid aggregates (red dotted lines) in human HCC specimens are composed of CD3+ T cells, CD20+ B cells, CD21+ FDCs, CD23+ GC cells, and DC-LAMP+ mature DCs (arrow). Scale bar, 100 μm. HCC, hepatocellular carcinoma; T, tumor tissue; P, peritumoral tissue; iTLS, intratumoral tertiary lymphoid structure; pTLS, peritumoral tertiary lymphoid structure; FDC, follicular dendritic cell; GC, germinal center; DC, dendritic cell.

The presence and location of TLS was initially assessed in H&E-stained sections of 240 HCC patients from West China Hospital. TLS was found within tumor tissue (iTLS) or peritumoral region (pTLS) or distant normal liver parenchymal (**Figure 1A**). iTLS presented in 76 (31.7%) tumors, whereas pTLS were detected in tumor periphery of 228 (95%) patients (**Figures 1B, C**). We also examined the presence of iTLS relative to pTLS density and found a higher pTLS density in cases presence of iTLS (**Figure 1D**). Histologically, the TLS contained B cell lymphoid follicles with or without germinal centers, T cell zones, and dendritic cells (DCs), which were arranged in a compartment similar to the organization of a lymph node (**Figure 1E**).

### The Density of pTLS Predicts Survival

**Figure 2:**
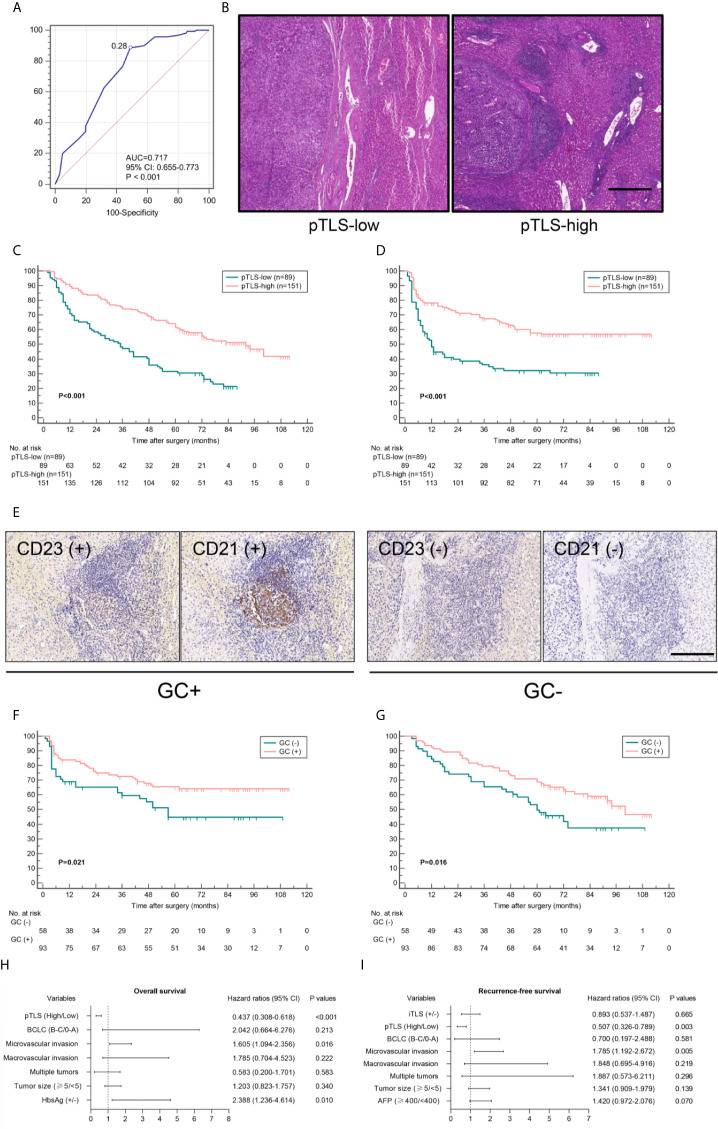
The density of pTLS correlates with patient survival outcomes. **(A)**, ROC curve analysis was used for identification of optimal cut-off value of pTLS density to separate pTLS-low and pTLS-high tumors with prognostic relevance. **(B)** Representative images of pTLS in pTLS-low group (<0.28 pTLS/mm^2^, left panel) and pTLS-high group (right panel) in H&E stained sections. Scale bar, 500 μm. **(C, D)** Kaplan-Meier curves were used for comparison of OS and RFS among patients with low or high pTLS density in training cohort. Significance was tested by log-rank test. **(E)** Representative images of GC (+) TLS (left) and GC (-) TLS in human HCC specimens by staining with CD21 and CD23. Scale bar, 50 μm. **(F, G)** Kaplan-Meier curves for OS and RFS in 151 patients with high pTLS density, according to the presence of GC or not. Significance was tested by log-rank test. **(H, I)** Multivariate analyses were performed to identify prognostic factors of OS and RFS in 240 HCC patients. pTLS, peritumoral tertiary lymphoid structure; ROC, receiver operating characteristics; AUC, area under curve; OS, overall survival; RFS, recurrence-free survival; GC, germinal center.

To investigate the prognostic effect of pTLS density on HCC patients, a threshold for separating patients with low and high pTLS densities was examined by using ROC curve (**Figures 2A, B**). A minimal p-value approach was used to test the determined cut-off and revealed that minimum p value was obtained at 0.28 pTLS/mm^2^ (**Supplementary Figure 1**). Kaplan-Meier survival analyses revealed that high pTLS density (> 0.28 pTLS/mm^2^, n=151) significantly correlated with improved OS and RFS in 240 HCC patients from West China Hospital and 120 patients from the Third Affiliated Hospital of Sun Yat-sen University (**Figures 2C, D** and **Supplementary Figure 2**). The presence of characteristic morphology of GC was observed in 106 (44.2%) tumors, showing improved survival outcomes than those without GC (**Figure 2E**, **Supplementary Figure 3**). In tumors with high pTLS densities, the presence of GC still correlated with better OS and RFS, suggesting its association with the best prognostic outcomes (**Figures 2F, G**). Univariate analyses demonstrated a significant correlation between high pTLS density and longer OS (hazard ratio, 0.418; 95% CI, 0.298-0.585) and RFS (hazard ratio, 0.437; 95% CI, 0.306-0.625, **Table 1**). Among all the significant covariates in univariate analyses, the pTLS density and microvascular invasion (MVI) were the only two independent prognostic factors of both OS and RFS in multivariate Cox regression analyses (**Figures 2H, I** and **Supplementary Table 4**). In addition, the positive association between high pTLS density and improved OS and RFS was confirmed by the validation cohort with geographically distinct HCC patients (**Supplementary Table 5**).

**Figure 3:**
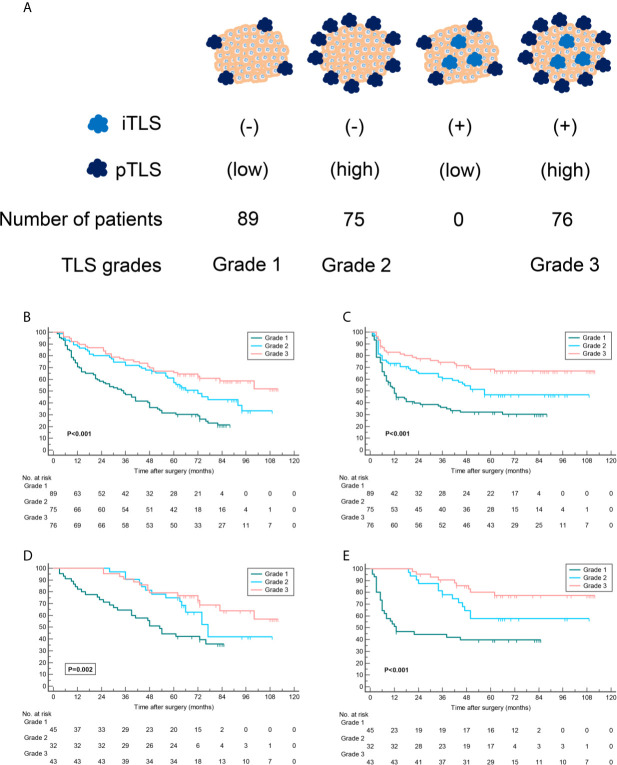
TLS grades correlate with patient survival outcomes. **(A)** Tumors were stratified according to the presence of iTLS and density of pTLS. Given no patient was associated with low pTLS density and presence of iTLS simultaneously, tumors were stratified into three grades. **(B–E)** Kaplan-Meier curves were used for comparison of OS and RFS among patients stratified by TLS grades in training cohort **(B, C)** and validation cohort **(D, E)**. Significance was tested by log-rank test. iTLS, intratumoral tertiary lymphoid structure; pTLS, peritumoral tertiary lymphoid structure; OS, overall survival; RFS, recurrence-free survival.

We next classified tumors into four categories to investigate prognostic significance of pTLS jointly with iTLS in HCC patients. The results shown that 89 (37.1%) tumors with iTLS absent and low pTLS density were classified as grade 1, 75 (31.2%) with iTLS absent and high pTLS density as grade 2, and 76 (31.7%) with iTLS present and high pTLS density as grade 3 (**Figure 3A**). No tumors with iTLS present and low pTLS density was found in the entire cohort. Further Kaplan-Meier curve analyses revealed that patients in grade 1 were associated with worst OS and RFS, whereas patients in grade 3 correlated with best prognosis, suggesting a possibly synergistic effect of pTLS and iTLS in predicting survival of HCC patients (**Figures 3B, C**). The findings were confirmed by the validation cohort of geographically distinct HCC patients (**Figures 3D, E**).

### Association Between pTLS and Clinicopathologic Features and Immune Microenvironment

**Figure 4:**
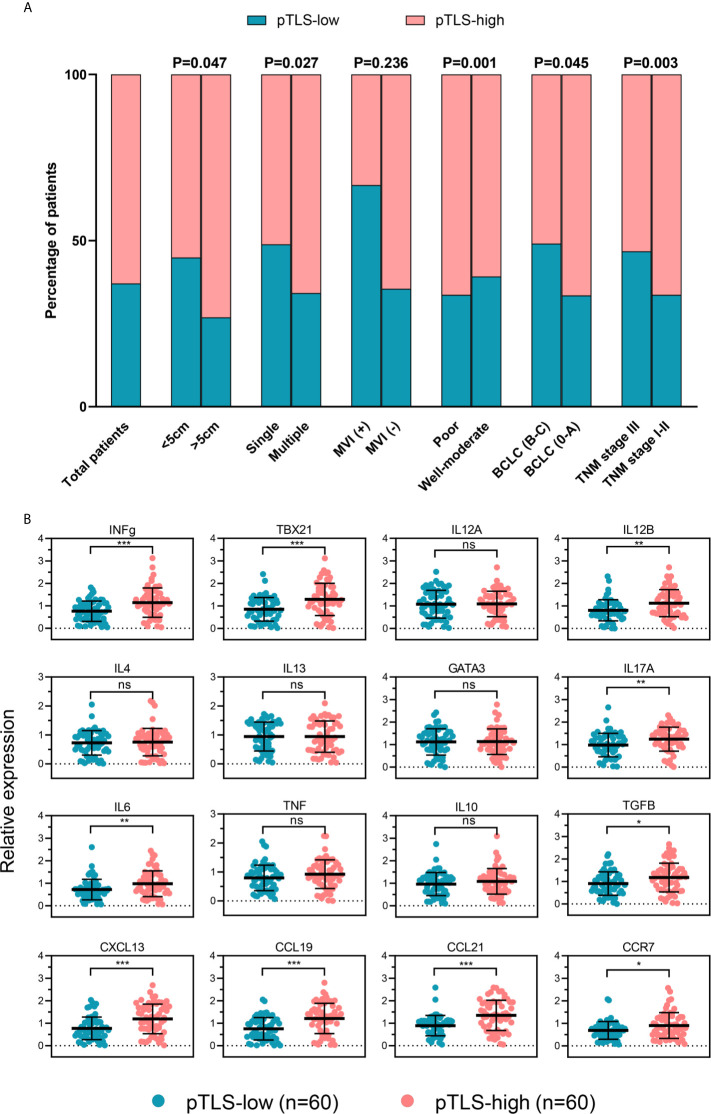
The density of pTLS correlates with clinicopathological features and tumor immune microenvironment. **(A)** pTLS-low or pTLS-high tumors in total HCC patients and in groups of patients stratified by clinicopathological features. **(B)** Expression of genes related to tumor immune microenvironment in HCC tissues of low pTLS (n=60) and high pTLS density (n=60), analyzed by real-time PCR. Groups were statistically compared using Mann–Whitney U-test. *P < 0.05; **P < 0.01; ***P < 0.001; ns, not significant. HCC, hepatocellular carcinoma; pTLS, peritumoral tertiary lymphoid structure; IHC, immunochemistry.

Correlations between pTLS and clinicopathological characteristics were further analyzed. Higher numbers of patients with high pTLS density were detected among those with lower TNM stages, lower BCLC stages, absence of MVI, solitary tumor and smaller tumor size (**Figure 4A**), suggesting a correlation between pTLS density with better prognosis.

To elucidate the possible reasons of prognostic benefit, we compared the gene expression profiles in HCC tissues from a set of patients with high pTLS density or low pTLS density by real-time PCR (**Figure 4B**). The baseline characteristics of patients were shown in **Supplementary Table 6**. As shown in **Supplementary Tables 7** and **8**, the clinicopathologic characteristics were comparable betweenwfi 2 frozen and FFPE HCC samples.Th1-orientation genes (INFg, TBX21 and IL12B) and Th17-associated genes IL17A were significantly upregulated in pTLS-high HCC tissues compared to those with pTLS-low, whereas the expression of IL12A was comparable between the groups. The expression of Th2-orientation genes IL4, IL13 and GATA3, were similar in both groups. Not all inflammation-related genes and immune suppression-related genes, but IL6 and TGFB, showed significantly upregulation in pTLS-high HCC tissues. We also examined the expression of the relevant chemokines in HCC tissues and found that CXCL13, CCL19 and CCL21 were higher expressed in pTLS-high tumors, compared to those with low pTLS densities. Additionally, the expression level of CCR7 was significantly upregulated in pTLS-high group.

### pTLS Facilitates Tumor-Infiltrating Immune Cells

**Figure 5:**
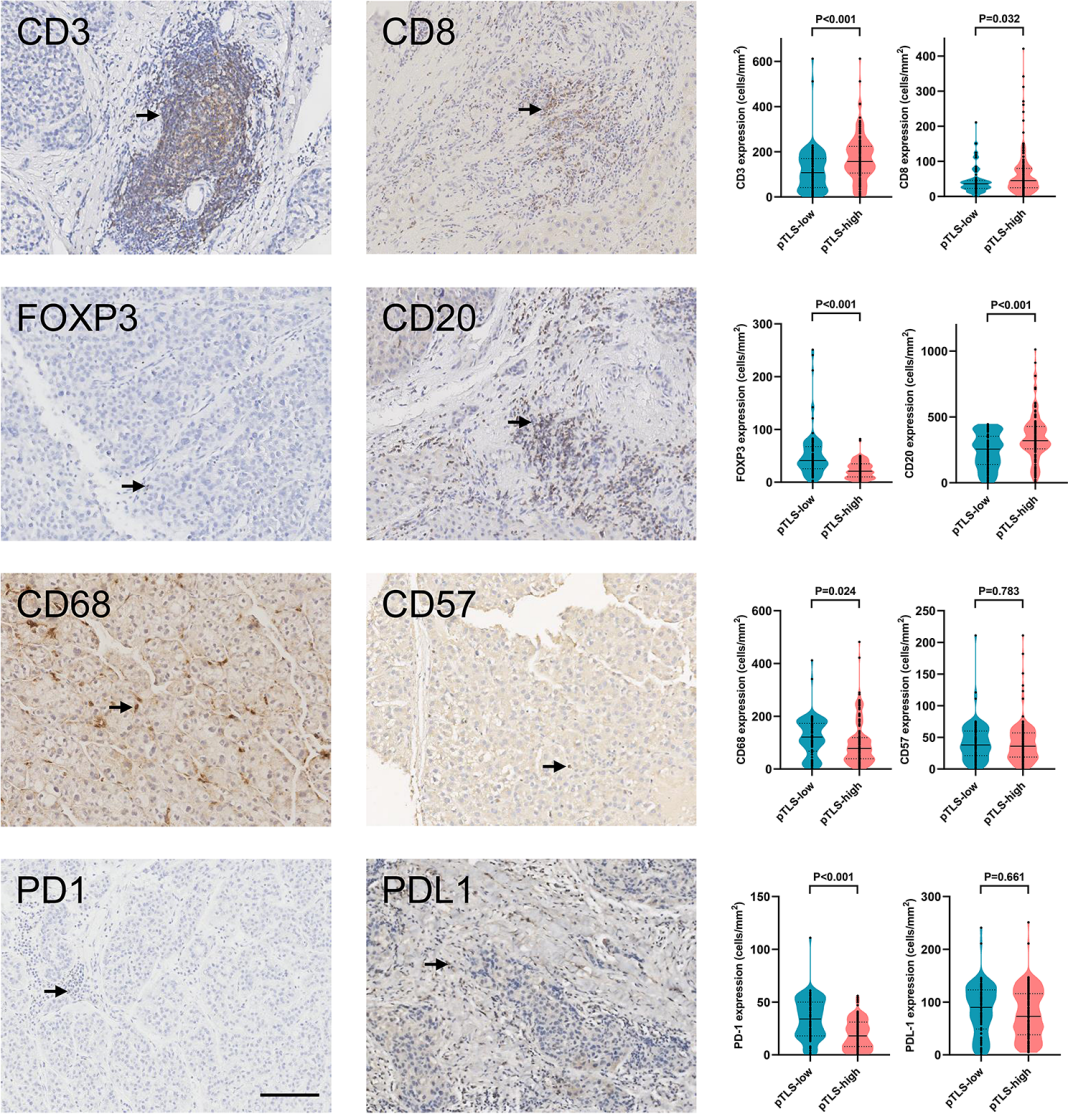
pTLS density correlates with tumor-infiltrating immune cells. Representative images of main tumor-infiltrated immune cells (left panel) and relative statistical analyses (right panel) of tumors with low or high pTLS densities. Black arrows represented positive staining. Groups were statistically compared using Mann–Whitney U-test. Scale bar, 100 μm. iTLS, intratumoral tertiary lymphoid structure; pTLS, peritumoral tertiary lymphoid structure.

We have previously revealed that presence of intratumoral TLS correlated with multiple tumour-infiltrating immune cells (20). We also examined the tumor-infiltrating immune cells in HCC tissues by immunochemistry (**Supplementary Figure 4**). In addition, we determined the association between pTLS density and tumor-infiltrating immune cells. pTLS-high tumors were significantly associated with increased intratumoral CD3+ T cells, CD8+ T cells, and CD20+ B cells, as compared to pTLS-low tumors (**Figure 5**). Reduced Foxp3+ regulatory T cells and CD68+ macrophages were infiltrated in pTLS-high tumors (**Figure 5**). These results were consistent with previous studies (20, 23, 24). The number of tumor-infiltrating CD57+ cells was similar between pTLS-high and pTLS-low tumors (**Figure 5**). We also evaluated PD1+ and PD-L1 cells in HCC tissues and found a significantly negative correlation between pTLS density with PD1+ cells (**Figure 5**). No significant association was observed between pTLS density and tumor-infiltrating PD-L1 cells (**Figure 5**). Additionally, we compared the cellular compositions of pTLS and iTLS roughly. As shown in **Supplementary Figure 5**, The median percentage of CD20 and CD68 were slightly lower in iTLS, whereas CD8 and CD57 were lower in pTLS. Furthermore, we compared the cellular composition of TLS between tumors with high pTLS and low density. The median percentages of infiltrating CD3, CD8, CD20 and CD57 were higher in high pTLS density TLS than those of low density, whereas FOXP3 and CD68 were higher in pTLS low group.

## Discussion

In the present study, we performed a comprehensive analysis of 360 cases of HCC tissues, and identified two different localizations of HCC-associated TLS, TLS intratumorally and peritumorally. The presence of iTLS was observed in 31.7% tumors, whereas pTLS almost ubiquitous (95%). Kaplan-Meier curve analyses showed high pTLS density alone or jointly with iTLS correlated with favorable clinical outcomes. Univariate and multivariate analyses identified the pTLS as independent prognostic factor for both OS and RFS. By analyses of tumor cytokine milieu and tumor-infiltrating immune cells, we revealed pTLS correlated with an active immune response in tumor microenvironment in HCC, which was similar to previous findings of breast cancer (25), lung cancer (9), colorectal cancer (26) and pancreatic cancer (11). To our knowledge, this is the first study that investigates the prognostic significance of peritumoral TLS in HCC and its correlation with tumor microenvironment.

TLS varied from simple lymphoid aggregate to complicated structures, composing of a follicular B cell zone, T cell-rich zone, mature DCs and HEVs. The formation of germinal center has been used to determine the maturation of TLS, stratifying TLS into different stages. Silina and colleagues demonstrated that the GC formation reflected the role of TLS in antitumor immunity, significantly relevant for patient survival and expression of several adaptive immune response-related genes in lung cancer (9). The prognostic significance of presence of GC was also evaluated in HCC, showing significant correlation with improved survival of HCC patients. In our work, 44% of tumors exhibiting characteristic morphology of GC, showed the best prognostic outcomes. A network of follicular dendritic cells located inside the GC could be detected by CD21 or CD23 labeling. HEVs are located at the periphery of TLS, allowing the entry of naïve and central-memory lymphocytes into TLS. Additionally, they functioned intratumorally as major gateways for circulating lymphocytes infiltrating into tumor centers (25). Apart from these components, TLS-associated regulatory T cells might help tumor escape by inhibiting endogenous immune responses against tumors (27).

We observed that the peritumoral TLS density significantly correlated with expression of CXCL13, CCL19 and CCL21 in HCC tissues. Previous studies have reported the crucial role of CXCL13 in follicular helper T cells and B cells recruitment, and intratumora CXCL13-producing CD4+ follicular helper T cells were relevant for prognosis in breast cancer (28). In addition, CXCL13 produced by TLS-associated perivascular cells served as entry sites for circulating lymphocytes into TLS (9). In this work, we also observed an increased CD20+ B cells infiltration in HCC with high peritumoral TLS densities. However, the mechanisms for perivascular cells actuating the expression of CXCL13 remain unclear. CCL19 and CCL21 are also required for lymphocyte recruitment, contributing to TLS development (29, 30). The intratumoral injection of CCL21 gene-modified DCs were found to facilitate eliciting systemic tumor-specific immune responses and tumor-infiltrating CD8+ T cells in lung cancer (30). In this study, we found an elevated chemokine receptor CCR7 expression level in pTLS high tumors. The CCR7 plays a critical role in lymphocyte and dendritic cell trafficking into and within lymph nodes (15). The increased intratumoral CCR7 expression favors lymphoid aggregating and TLS formation. However, CCR7 could also be expressed by the tumor cells themselves. A number of studies have demonstrated the pro-tumoral effect of CCR7 in several carcinomas including HCC (31–33). Recent study has revealed that chemokine receptor like 1 (CCRL1), a member of atypical chemokine receptors, could sequestrate CCL19 and CCL21, and thereby reduce their binding to CCR7 (34). CCRL1 mitigated the detrimental impact of CCR7 in the progression and metastasis of HCC. The potential link between CCR7 and pTLS as well as prognostic value in HCC progression are still needed further investigation.

In the present study, an elevated IL-6 expression was observed in tumors with pTLS high densities. This was consistent with a recent study which revealed that IL-6 was higher expressed in pancreatic cancer tissues with iTLS compared to those without (11). The upregulation of IL-6 could accelerate the differentiation of Th17 and inhibit the differentiation of Tregs into stable immune inhibitory cells, facilitating an active immune microenvironment (35). However, aberrant IL-6 expression also played an important role in tumorigenesis and cancer progression *via* promoting tumor cell growth and metastasis (36, 37). Previous reports have described that the presence of IL-6 impeded lymphocyte-dendritic cell cross-talk (38). Additionally, IL-6 signaling activated the *in vivo* growth of HCC progenitor cells and malignant progression (39). Further studies are needed to confirm our results.

To gain better understand the association between peritumoral TLS density with patient survival, we assessed the expression of tumor immune-related genes and intratumoral infiltrating immune cells. The pTLS-high density tumors highly expressed sets of gene characteristics of Th1-oriention and immunosuppression, suggesting an active immune response in tumor microenvironment. The Th17-oriented gene IL17A were also significantly overexpressed in tumors with high peritumoral TLS density. Although the prognostic significance of Th17 infiltration remains controversial in various of malignancies (4, 40–42). Wang and colleagues revealed IL17A promoted tumor progression *via* STAT3/NF-κB/Notch1 pathway. Given Th17 cells share several developmental and effector markers with lymphoid-tissue inducer cells and their related innate lymphoid cells, it is possible for Th17-related cells contribute to antitumor immune responses directly or *via* induction of intratumoral TLS (43). However, the underlying mechanisms remain unknown.

The favorable impact of tumor-infiltrating cytotoxic T cells on clinical outcomes has been evaluated in various cancers, including lung cancer (44), colon tumors (45), and ovarian cancer (46). Thus, the correlation between high peritumoral TLS density with improved survival could be partly explained by high density of tumor-infiltrating CD8+ T cells in HCC patients. Furthermore, high peritumoral TLS densities were associated with high numbers of B cells infiltration within tumor. B cells were considered actively participate in immune responses *via* directing T cell response to antigens (47). In contrast, lower densities of CD68+ macrophages and Foxp3+ Treg cells were detected in tumors with high pTLS densities, consistently with our previous study (20). Infiltration of Treg cells have been showed relative to poor patient prognosis in various tumor types (48, 49). In a recent study by Joshi et al. Treg cell depletion increased expression of costimulatory ligands by expansion of DCs, leading to tumor destruction in a genetically-engineered mouse lung adenocarcinoma model (27).

Our results identified pTLS density as the most significant and independent prognostic indicator for OS and RFS in surgically treated HCC patients, outperforming tumor stages and MVI.

In summary, we demonstrate that high pTLS density correlates with improved clinical outcomes and appears to be an independent prognosticator in HCC. Our findings also reveal an interplay between the infiltrated immune cells within the intratumoral microenvironment, reflecting an active immune reaction, and pTLS. Thus, this study provides strong evidence supporting the idea that tumor immune microenvironment components markedly correlated with patient clinical outcomes. High pTLS density links to a tumor microenvironment with an active immune reaction and improved patient survival and represents a promising prognostic biomarker for HCC. Further studies focusing on mechanisms leading to TLS formation, immune cell recruitment and generate antitumor immune activation are in need, which provide opportunities for developing TLS-targeted immunomodulatory therapies.

## Data Availability Statement

The original contributions presented in the study are included in the article/**Supplementary Material**. Further inquiries can be directed to the corresponding authors.

## Ethics Statement

The studies involving human participants were reviewed and approved by Institutional Review Board of West China Hospital of Sichuan University Institutional Review Board of the Third Affiliated Hospital of Sun Yat-sen University. The patients/participants provided their written informed consent to participate in this study.

## Author Contributions

Conception and designation: HW, GW, and HL. Clinical data collection: HL, HLL, and JL. Data analysis and drafting the manuscript: HL, HLL, and HF. Statistical analysis: HL and HLL. Technical support: HLL and LX. All authors contributed to the article and approved the submitted version.

## Funding

This work was supported by National Natural Science Foundation of China (81972747, 81872004, 81800564, 81770615, 81700555 and 81672882), the Science and Technology Support Program of Sichuan Province (2021YFH0095, 2019YFQ0001, 2018SZ0115, 2017SZ0003), the Science and Technology Program of Tibet Autonomous Region (XZ201801-GB-02) and the 1.3.5 project for disciplines of excellence, West China Hospital, Sichuan University (ZYJC18008).

## Conflict of Interest

The authors declare that the research was conducted in the absence of any commercial or financial relationships that could be construed as a potential conflict of interest.

## Abbreviations

HCC, hepatocellular carcinoma; TLS, tertiary lymphoid structure; pTLS, peritumoral TLS; iTLS, intratumoral TLS; GC, germinal center; TIL, tumor-infiltrating lymphocytes; HEVs, high endothelial venules; DCs, dendritic cells; OS, Overall survival; RFS, Recurrence-free survival; WHO, World Health Organization; TNM, tumor-node-metastasis staging system; H&E, hematoxylin and eosin; FFPE, formalin-fixed, paraffin-embedded; ROC, operating characteristics.

## References

[1] Bray F Ferlay J Soerjomataram I Siegel RL Torre LA Jemal A. Global Cancer Statistics 2018: GLOBOCAN Estimates of Incidence and Mortality Worldwide for 36 Cancers in 185 Countries. CA Cancer J Clin (2018) 68(6):394–424. 10.3322/caac.21492 30207593

[2] Llovet JM Zucman-Rossi J Pikarsky E Sangro B Schwartz M Sherman M. Hepatocellular Carcinoma. Nat Rev Dis Primers (2016) 2:16018. 10.1038/nrdp.2016.18 27158749

[3] Keren L Bosse M Marquez D Angoshtari R Jain S Varma S. A Structured Tumor-Immune Microenvironment in Triple Negative Breast Cancer Revealed by Multiplexed Ion Beam Imaging. Cell (2018) 174(6):1373–87.e19. 10.1016/j.cell.2018.08.039 30193111 PMC6132072

[4] Fridman WH Pages F Sautes-Fridman C Galon J. The Immune Contexture in Human Tumours: Impact on Clinical Outcome. Nat Rev Cancer (2012) 12(4):298–306. 10.1038/nrc3245 22419253

[5] Di Caro G Bergomas F Grizzi F Doni A Bianchi P Malesci A. Occurrence of Tertiary Lymphoid Tissue is Associated With T-cell Infiltration and Predicts Better Prognosis in Early-Stage Colorectal Cancers. Clin Cancer Res (2014) 20(8):2147–58. 10.1158/1078-0432.ccr-13-2590 24523438

[6] Dieu-Nosjean MC Antoine M Danel C Heudes D Wislez M Poulot V. Long-Term Survival for Patients With Non-Small-Cell Lung Cancer With Intratumoral Lymphoid Structures. J Clin Oncol (2008) 26(27):4410–7. 10.1200/jco.2007.15.0284 18802153

[7] Engelhard VH Rodriguez AB Mauldin IS Woods AN Peske JD Slingluff CL Jr. Immune Cell Infiltration and Tertiary Lymphoid Structures as Determinants of Antitumor Immunity. J Immunol (2018) 200(2):432–42. 10.4049/jimmunol.1701269 PMC5777336 29311385

[8] Dieu-Nosjean MC Goc J Giraldo NA Sautes-Fridman C Fridman WH. Tertiary Lymphoid Structures in Cancer and Beyond. Trends Immunol (2014) 35(11):571–80. 10.1016/j.it.2014.09.006 25443495

[9] Silina K Soltermann A Attar FM Casanova R Uckeley ZM Thut H. Germinal Centers Determine the Prognostic Relevance of Tertiary Lymphoid Structures and Are Impaired by Corticosteroids in Lung Squamous Cell Carcinoma. Cancer Res (2018) 78(5):1308–20. 10.1158/0008-5472.Can-17-1987 29279354

[10] de Chaisemartin L Goc J Damotte D Validire P Magdeleinat P Alifano M. Characterization of Chemokines and Adhesion Molecules Associated With T Cell Presence in Tertiary Lymphoid Structures in Human Lung Cancer. Cancer Res (2011) 71(20):6391–9. 10.1158/0008-5472.Can-11-0952 21900403

[11] Hiraoka N Ino Y Yamazaki-Itoh R Kanai Y Kosuge T Shimada K. Intratumoral Tertiary Lymphoid Organ Is a Favourable Prognosticator in Patients With Pancreatic Cancer. Br J Cancer (2015) 112(11):1782–90. 10.1038/bjc.2015.145 PMC4647237 25942397

[12] Goc J Germain C Vo-Bourgais TK Lupo A Klein C Knockaert S. Dendritic Cells in Tumor-Associated Tertiary Lymphoid Structures Signal a Th1 Cytotoxic Immune Contexture and License the Positive Prognostic Value of Infiltrating Cd8+ T Cells. Cancer Res (2014) 74(3):705–15. 10.1158/0008-5472.can-13-1342 24366885

[13] Yeh JC Hiraoka N Petryniak B Nakayama J Ellies LG Rabuka D. Novel Sulfated Lymphocyte Homing Receptors and Their Control by a Core1 Extension Beta 1,3-N-Acetylglucosaminyltransferase. Cell (2001) 105(7):957–69. 10.1016/s0092-8674(01)00394-4 11439191

[14] Cabrita R Lauss M Sanna A Donia M Skaarup Larsen M Mitra S. Tertiary Lymphoid Structures Improve Immunotherapy and Survival in Melanoma. Nature (2020) 577(7791):561–5. 10.1038/s41586-019-1914-8 31942071

[15] Dieu-Nosjean MC Giraldo NA Kaplon H Germain C Fridman WH Sautès-Fridman C. Tertiary Lymphoid Structures, Drivers of the Anti-Tumor Responses in Human Cancers. Immunol Rev (2016) 271(1):260–75. 10.1111/imr.12405 27088920

[16] Liu X Tsang JYS Hlaing T Hu J Ni YB Chan SK. Distinct Tertiary Lymphoid Structure Associations and Their Prognostic Relevance in HER2 Positive and Negative Breast Cancers. Oncologist (2017) 22(11):1316–24. 10.1634/theoncologist.2017-0029 PMC5679825 28701569

[17] Carrega P Loiacono F Di Carlo E Scaramuccia A Mora M Conte R. Ncr(+)Ilc3 Concentrate in Human Lung Cancer and Associate With Intratumoral Lymphoid Structures. Nat Commun (2015) 6:8280. 10.1038/ncomms9280 26395069

[18] Finkin S Yuan D Stein I Taniguchi K Weber A Unger K. Ectopic Lymphoid Structures Function as Microniches for Tumor Progenitor Cells in Hepatocellular Carcinoma. Nat Immunol (2015) 16(12):1235–44. 10.1038/ni.3290 PMC4653079 26502405

[19] Calderaro J Petitprez F Becht E Laurent A Hirsch TZ Rousseau B. Intra-Tumoral Tertiary Lymphoid Structures Are Associated With a Low Risk of Early Recurrence of Hepatocellular Carcinoma. J Hepatol (2019) 70(1):58–65. 10.1016/j.jhep.2018.09.003 30213589

[20] Li H Wang J Liu H Lan T Xu L Wang G. Existence of Intratumoral Tertiary Lymphoid Structures Is Associated With Immune Cells Infiltration and Predicts Better Prognosis in Early-Stage Hepatocellular Carcinoma. Aging (Albany NY) (2020) 12(4):3451–72. 10.18632/aging.102821 PMC7066901 32087064

[21] Calderaro J Petitprez F Becht E Laurent A Hirsch TZ Rousseau B. Intra-Tumoral Tertiary Lymphoid Structures are Associated With a Low Risk of Early Recurrence of Hepatocellular Carcinoma. J Hepatol (2018) 70(1):58–65. 10.1016/j.jhep.2018.09.003 30213589

[22] Wei L Delin Z Kefei Y Hong W Jiwei H Yange Z. A Classification Based on Tumor Budding and Immune Score for Patients With Hepatocellular Carcinoma. Oncoimmunology (2020) 9(1):1672495. 10.1080/2162402x.2019.1672495 32002283 PMC6959452

[23] Steele KE Brown C. Multiplex Immunohistochemistry for Image Analysis of Tertiary Lymphoid Structures in Cancer. Methods Mol Biol (2018) 1845:87–98. 10.1007/978-1-4939-8709-2_6 30141009

[24] Behr DS Peitsch WK Hametner C Lasitschka F Houben R Schönhaar K. Prognostic Value of Immune Cell Infiltration, Tertiary Lymphoid Structures and PD-L1 Expression in Merkel Cell Carcinomas. Int J Clin Exp Pathol (2014) 7(11):7610–21. PMC4270630 25550797

[25] Martinet L Garrido I Filleron T Le Guellec S Bellard E Fournie JJ. Human Solid Tumors Contain High Endothelial Venules: Association With T- and B-lymphocyte Infiltration and Favorable Prognosis in Breast Cancer. Cancer Res (2011) 71(17):5678–87. 10.1158/0008-5472.can-11-0431 21846823

[26] Posch F Silina K Leibl S Mundlein A Moch H Siebenhuner A. Maturation of Tertiary Lymphoid Structures and Recurrence of Stage II and III Colorectal Cancer. Oncoimmunology (2018) 7(2):e1378844. 10.1080/2162402x.2017.1378844 29416939 PMC5798199

[27] Joshi NS Akama-Garren EH Lu Y Lee DY Chang GP Li A. Regulatory T Cells in Tumor-Associated Tertiary Lymphoid Structures Suppress Anti-Tumor T Cell Responses. Immunity (2015) 43(3):579–90. 10.1016/j.immuni.2015.08.006 PMC4826619 26341400

[28] Gu-Trantien C Loi S Garaud S Equeter C Libin M de Wind A. Cd4(+) Follicular Helper T Cell Infiltration Predicts Breast Cancer Survival. J Clin Invest (2013) 123(7):2873–92. 10.1172/jci67428 PMC3696556 23778140

[29] Kuroda E Ozasa K Temizoz B Ohata K Koo CX Kanuma T. Inhaled Fine Particles Induce Alveolar Macrophage Death and Interleukin-1alpha Release to Promote Inducible Bronchus-Associated Lymphoid Tissue Formation. Immunity (2016) 45(6):1299–310. 10.1016/j.immuni.2016.11.010 28002730

[30] Hwang JY Randall TD Silva-Sanchez A. Inducible Bronchus-Associated Lymphoid Tissue: Taming Inflammation in the Lung. Front Immunol (2016) 7:258. 10.3389/fimmu.2016.00258 27446088 PMC4928648

[31] Yang L Chang Y Cao P. Ccr7 Preservation Via Histone Deacetylase Inhibition Promotes Epithelial-Mesenchymal Transition of Hepatocellular Carcinoma Cells. Exp Cell Res (2018) 371(1):231–7. 10.1016/j.yexcr.2018.08.015 30107147

[32] An S Tiruthani K Wang Y Xu L Hu M Li J. Locally Trapping the C-C Chemokine Receptor Type 7 by Gene Delivery Nanoparticle Inhibits Lymphatic Metastasis Prior to Tumor Resection. Small (2019) 15(9):e1805182. 10.1002/smll.201805182 30690891 PMC6878664

[33] Sancho M Vieira JM Casalou C Mesquita M Pereira T Cavaco BM. Expression and Function of the Chemokine Receptor CCR7 in Thyroid Carcinomas. J Endocrinol (2006) 191(1):229–38. 10.1677/joe.1.06688 17065406

[34] Shi JY Yang LX Wang ZC Wang LY Zhou J Wang XY. Cc Chemokine Receptor-Like 1 Functions as a Tumour Suppressor by Impairing CCR7-Related Chemotaxis in Hepatocellular Carcinoma. J Pathol (2015) 235(4):546–58. 10.1002/path.4450 25255875

[35] Longhi MS Liberal R Holder B Robson SC Ma Y Mieli-Vergani G. Inhibition of Interleukin-17 Promotes Differentiation of CD25⁻ Cells Into Stable T Regulatory Cells in Patients With Autoimmune Hepatitis. Gastroenterology (2012) 142(7):1526–35.e6. 10.1053/j.gastro.2012.02.041 22387392

[36] Chan LC Li CW Xia W Hsu JM Lee HH Cha JH. Il-6/Jak1 Pathway Drives PD-L1 Y112 Phosphorylation to Promote Cancer Immune Evasion. J Clin Invest (2019) 129(8):3324–38. 10.1172/jci126022 PMC6668668 31305264

[37] Ye X Wu H Sheng L Liu YX Ye F Wang M. Oncogenic Potential of Truncated Rxrα During Colitis-Associated Colorectal Tumorigenesis by Promoting Il-6-STAT3 Signaling. Nat Commun (2019) 10(1):1463. 10.1038/s41467-019-09375-8 30931933 PMC6443775

[38] Cabillic F Bouet-Toussaint F Toutirais O Rioux-Leclercq N Fergelot P de la Pintière CT. Interleukin-6 and Vascular Endothelial Growth Factor Release by Renal Cell Carcinoma Cells Impedes Lymphocyte-Dendritic Cell Cross-Talk. Clin Exp Immunol (2006) 146(3):518–23. 10.1111/j.1365-2249.2006.03212.x PMC1810419 17100773

[39] He G Dhar D Nakagawa H Font-Burgada J Ogata H Jiang Y. Identification of Liver Cancer Progenitors Whose Malignant Progression Depends on Autocrine Il-6 Signaling. Cell (2013) 155(2):384–96. 10.1016/j.cell.2013.09.031 PMC4015514 24120137

[40] Quan H Shan Z Liu Z Liu S Yang L Fang X. The Repertoire of Tumor-Infiltrating Lymphocytes Within the Microenvironment of Oral Squamous Cell Carcinoma Reveals Immune Dysfunction. Cancer Immunol Immunother (2020) 69(3):465–76. 10.1007/s00262-020-02479-x PMC11027813 31950224

[41] Wang JT Li H Zhang H Chen YF Cao YF Li RC. Intratumoral IL17-producing Cells Infiltration Correlate With Antitumor Immune Contexture and Improved Response to Adjuvant Chemotherapy in Gastric Cancer. Ann Oncol (2019) 30(2):266–73. 10.1093/annonc/mdy505 30445581

[42] Aotsuka A Matsumoto Y Arimoto T Kawata A Ogishima J Taguchi A. Interleukin-17 is Associated With Expression of Programmed Cell Death 1 Ligand 1 in Ovarian Carcinoma. Cancer Sci (2019) 110(10):3068–78. 10.1111/cas.14174 PMC6778630 31432577

[43] Spits H Artis D Colonna M Diefenbach A Di Santo JP Eberl G. Innate Lymphoid Cells–a Proposal for Uniform Nomenclature. Nat Rev Immunol (2013) 13(2):145–9. 10.1038/nri3365 23348417

[44] Ganesan AP Clarke J Wood O Garrido-Martin EM Chee SJ Mellows T. Tissue-Resident Memory Features are Linked to the Magnitude of Cytotoxic T Cell Responses in Human Lung Cancer. Nat Immunol (2017) 18(8):940–50. 10.1038/ni.3775 PMC6036910 28628092

[45] Marisa L Svrcek M Collura A Becht E Cervera P Wanherdrick K. The Balance Between Cytotoxic T-Cell Lymphocytes and Immune Checkpoint Expression in the Prognosis of Colon Tumors. J Natl Cancer Inst (2018) 110(1):68–77. 10.1093/jnci/djx136 28922790

[46] Hamanishi J Mandai M Iwasaki M Okazaki T Tanaka Y Yamaguchi K. Programmed Cell Death 1 Ligand 1 and Tumor-Infiltrating Cd8+ T Lymphocytes are Prognostic Factors of Human Ovarian Cancer. Proc Natl Acad Sci USA (2007) 104(9):3360–5. 10.1073/pnas.0611533104 PMC1805580 17360651

[47] Lund FE Randall TD. Effector and Regulatory B Cells: Modulators of CD4+ T Cell Immunity. Nat Rev Immunol (2010) 10(4):236–47. 10.1038/nri2729 PMC3038334 20224569

[48] De Simone M Arrigoni A Rossetti G Gruarin P Ranzani V Politano C. Transcriptional Landscape of Human Tissue Lymphocytes Unveils Uniqueness of Tumor-Infiltrating T Regulatory Cells. Immunity (2016) 45(5):1135–47. 10.1016/j.immuni.2016.10.021 PMC5119953 27851914

[49] Liu XS Lin XK Mei Y Ahmad S Yan CX Jin HL. Regulatory T Cells Promote Overexpression of Lgr5 on Gastric Cancer Cells Via TGF-beta1 and Confer Poor Prognosis in Gastric Cancer. Front Immunol (2019) 10:1741. 10.3389/fimmu.2019.01741 31417548 PMC6682668

