# Supplementary figures and images for: Peritumoral Tertiary Lymphoid Structures Correlate With Protective Immunity and Improved Prognosis in Patients With Hepatocellular Carcinoma
Source: Front Immunol. 2021 May 26;12:648812. doi: 10.3389/fimmu.2021.648812 (PMC8187907; doi:10.3389/fimmu.2021.648812)

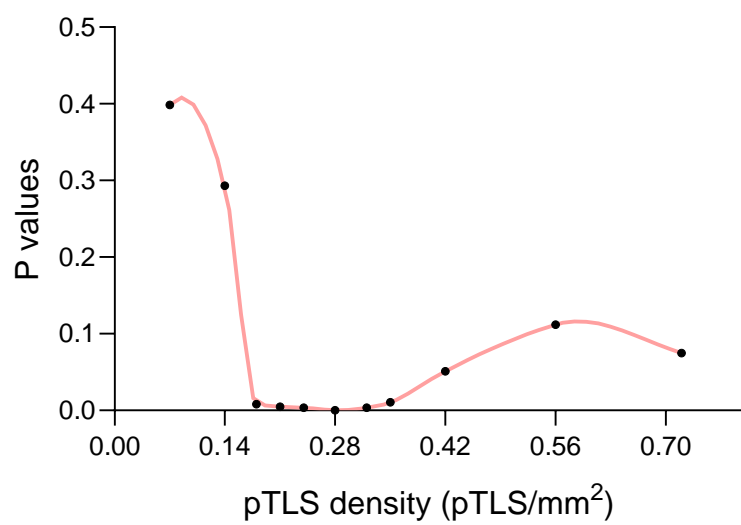

Supplement: Supplementary file 1 [file DataSheet_1.pdf]

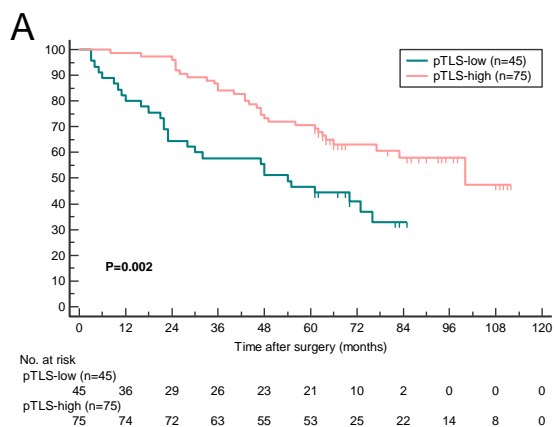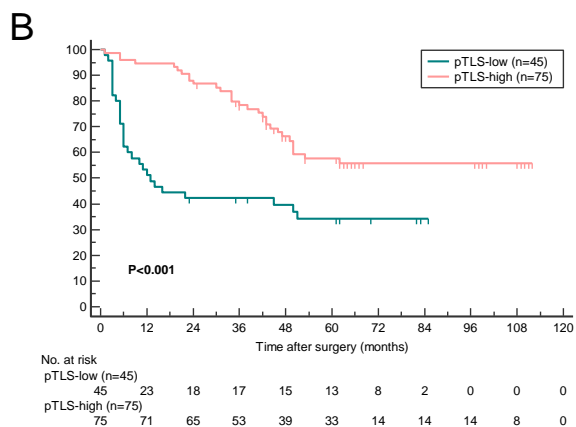

Supplement: Supplementary file 2 [file DataSheet_2.pdf]

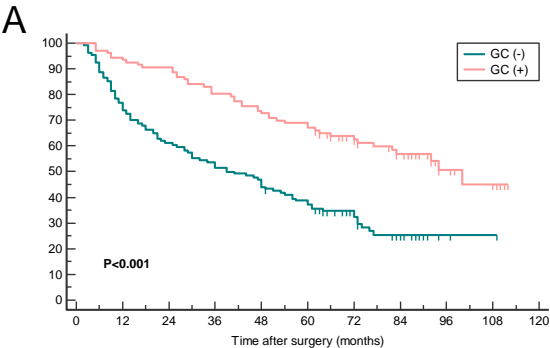

No. at risk

|        |     |    |    |    |    |    |    |    |    |   |   |
|--------|-----|----|----|----|----|----|----|----|----|---|---|
| GC (-) | 134 | 99 | 82 | 69 | 59 | 49 | 25 | 12 | 3  | 1 | 0 |
| GC (+) | 106 | 99 | 96 | 85 | 77 | 71 | 47 | 35 | 12 | 7 | 0 |

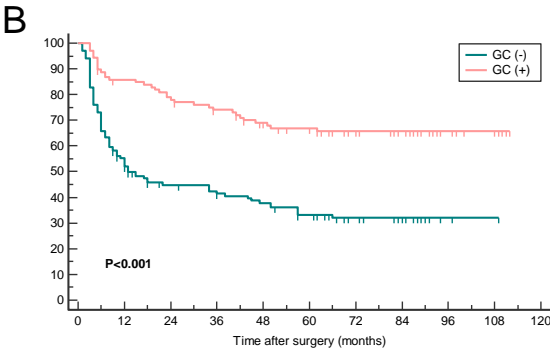

No. at risk

|        |     |    |    |    |    |    |    |    |    |   |   |
|--------|-----|----|----|----|----|----|----|----|----|---|---|
| GC (-) | 134 | 67 | 53 | 46 | 42 | 35 | 21 | 12 | 3  | 1 | 0 |
| GC (+) | 106 | 88 | 80 | 74 | 64 | 58 | 40 | 31 | 12 | 7 | 0 |

Supplement: Supplementary file 3 [file DataSheet_3.pdf]

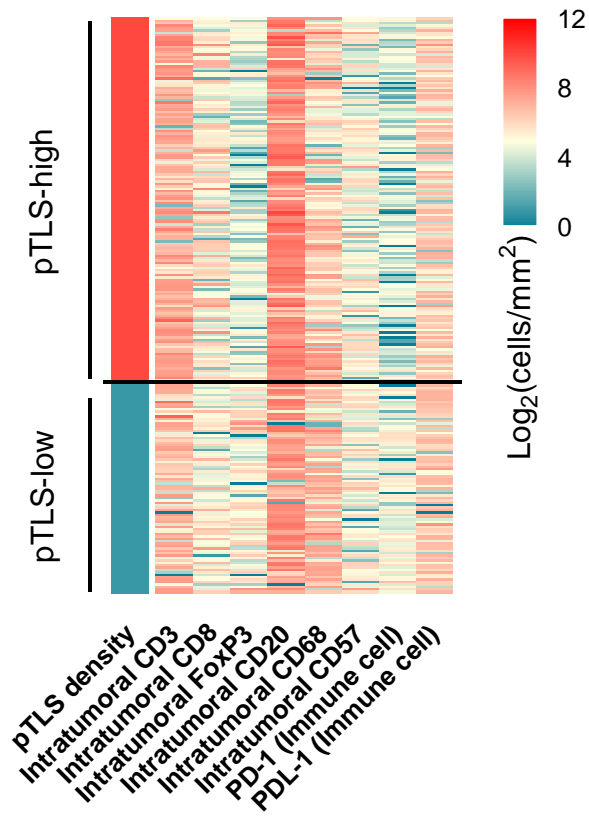

Supplement: Supplementary file 4 [file DataSheet_4.pdf]
