# Supplementary material for: Peritumoral Tertiary Lymphoid Structures Correlate With Protective Immunity and Improved Prognosis in Patients With Hepatocellular Carcinoma
Source: Front Immunol. 2021 May 26;12:648812. doi: 10.3389/fimmu.2021.648812 (PMC8187907; doi:10.3389/fimmu.2021.648812)
Supplement: Supplementary file 5 [file DataSheet_5.pdf]

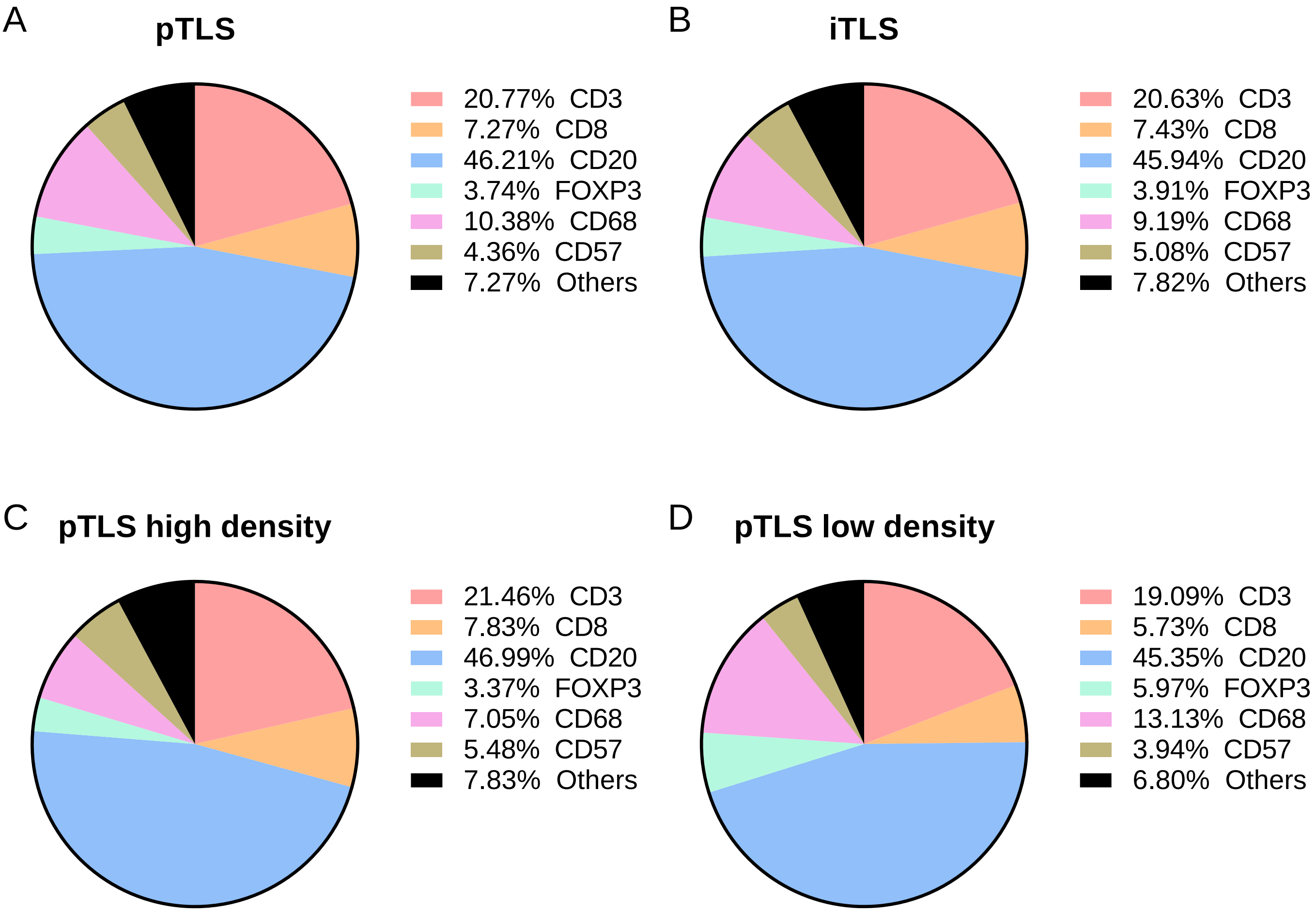
A
pTLS
20.77% CD3
7.27% CD8
46.21% CD20
3.74% FOXP3
10.38% CD68
4.36% CD57
7.27% Others
B
iTLS
20.63% CD3
7.43% CD8
45.94% CD20
3.91% FOXP3
9.19% CD68
5.08% CD57
7.82% Others
C
pTLS high density
21.46% CD3
7.83% CD8
46.99% CD20
3.37% FOXP3
7.05% CD68
5.48% CD57
7.83% Others
D
pTLS low density
19.09% CD3
5.73% CD8
45.35% CD20
5.97% FOXP3
13.13% CD68
3.94% CD57
6.80% Others
