# Supplementary material for: Peritumoral Tertiary Lymphoid Structures Correlate With Protective Immunity and Improved Prognosis in Patients With Hepatocellular Carcinoma
Source: Front Immunol. 2021 May 26;12:648812. doi: 10.3389/fimmu.2021.648812 (PMC8187907; doi:10.3389/fimmu.2021.648812)
Supplement: Supplementary file 6 [file Table_1.docx]

**Supplementary figure 1. A minimal P-value approach illustrates the optimal cut-off value of pTLS density for predicting the survival of 240 HCC patients.**

**Supplementary figure 2.** **The density of pTLS correlates with patient survival outcomes in validation cohort.** Kaplan-Meier curves for OS (A) and RFS (B) in 120 patients from validation cohort, according to the density of pTLS. Significance was tested by log-rank test. pTLS, peritumoral tertiary lymphoid structure; OS, overall survival; RFS, recurrence-free survival.

**Supplementary figure 3.** **The presence of GC correlates with patient survival outcomes.** Kaplan-Meier curves for OS (A) and RFS (B) in 240 patients from the West China Hospital, according to the presence of GC or not. Significance was tested by log-rank test. pTLS, peritumoral tertiary lymphoid structure; OS, overall survival; RFS, recurrence-free survival; GC, germinal center.

**Supplementary figure 4. Heat map showing tumor-infiltrating immune cells and their relevance to pTLS density in 240 HCC tissues.** IHC was used for evaluation of tumor-infiltrating immune cells. HCC, hepatocellular carcinoma; pTLS, peritumoral tertiary lymphoid structure; IHC, immunochemistry.

**Supplementary figure 5. Pie charts displayed the main cellular composition of TLS in 240 HCC patients.** Composition of pTLS (A) and iTLS (B); Cellular composition of main cell types within pTLS in tumors with high pTLS densities (C) and low pTLS densities (D). pTLS, peritumoral tertiary lymphoid structure; iTLS, intratumoral tertiary lymphoid structure; HCC, hepatocellular carcinoma.

Supplementary table 1. Comparing of baseline characteristics between patients from training cohort and validation cohort.

| Variables | Training cohort (n=240) | Validation cohort (n=120) | P value |
| --- | --- | --- | --- |
| Age (<50/≥50) | 117/123 | 58/62 | 1.000 |
| Gender (male/female) | 202/38 | 99/21 | 0.763 |
| HBsAg (+/-) | 211/29 | 103/17 | 0.617 |
| Cirrhosis (+/-) | 150/90 | 79/41 | 0.563 |
| Portal hypertension (+/-) | 36/204 | 17/103 | 0.876 |
| AFP, ng/dL (<400/≥400) | 139/101 | 77/43 | 0.304 |
| Tumor size, cm (<5/≥5) | 104/136 | 58/62 | 0.432 |
| Tumor number (single/ multiple) | 193/47 | 102/18 | 0.312 |
| Differentiation (well-moderate/poor) | 148/92 | 68/52 | 0.424 |
| Macrovascular invasion (+/-) | 12/228 | 7/113 | 0.804 |
| Microvascular invasion (+/-) | 81/159 | 37/83 | 0.634 |
| BCLC stages (0-A/B-C) | 185/55 | 102/18 | 0.095 |
| TNM stages (Ⅰ-Ⅱ/Ⅲ) | 178/62 | 98/22 | 0.146 |
| pTLS (low/high) | 89/151 | 45/75 | 1.000 |
| iTLS (-/+) | 164/76 | 82/38 | 1.000 |
| 5-year survival | 124 (51.7%) | 74 (61.7%) | 0.092 |

AFP: alpha-fetoprotein; BCLC: Barcelona Clinic Liver Cancer; TNM: tumor-nodes-metastasis; pTLS: peritumoral tertiary lymphoid structure; iTLS: intratumoral tertiary lymphoid structure; OS: overall survival; RFS: recurrence-free survival; HCC: hepatocellular carcinoma.

Supplementary table 2. Antibody sources and staining conditions.

| Markers | Antibody sources | Cellular location | Species | Antigen retrieval | Dilution |
| --- | --- | --- | --- | --- | --- |
| CD3 | Abcam, ab16669 | Membranous | Rabbit monoclonal | Citrate buffer (pH 6.0) | 1:200 |
| CD20 | Abcam, ab9475 | Membranous | Mouse monoclonal | Citrate buffer (pH 6.0) | 1:200 |
| CD21 | Abcam, ab75985 | Membranous | Rabbit monoclonal | Tris/EDTA buffer (pH 9.0) | 1:500 |
| CD23 | Abclonal, A11436 | Membranous | Rabbit monoclonal | Tris/EDTA buffer (pH 9.0) | 1:200 |
| DC LAMP | Abclonal, A2895 | Membranous | Rabbit polyclonal | Citrate buffer (pH 6.0) | 1:100 |
| CD8 | Abcam, ab33786 | Membranous | Mouse monoclonal | Citrate buffer (pH 6.0) | 1:200 |
| FOXP3 | Abcam, ab20034 | Nuclear | Mouse monoclonal | Citrate buffer (pH 6.0) | 1:200 |
| CD68 | DAKO, M087601-2 | Cytoplasmic | Mouse monoclonal | Citrate buffer (pH 6.0) | 1:200 |
| CD57 | Abcam, ab233871 | Membranous | Mouse monoclonal | Tris/EDTA buffer (pH 9.0) | 1:200 |
| PD1 | Abcam, ab52587 | Membranous | Mouse monoclonal | Citrate buffer (pH 6.0) | 1:50 |
| PDL1 | CST, #13684 | Membranous | Rabbit monoclonal | Tris/EDTA buffer (pH 9.0) | 1:200 |

Supplementary table 3. The primers used in this study.

| Genes | Forward | Reverse |
| --- | --- | --- |
| IFNg | ATGCAGGTCATTCAGATGTAGC | GACAATTTGGCTCTGCATTATTT |
| TBX21 | GACTCCCCCAACACAGGAG | GGGACTGGAGCACAATCATC |
| IL12A | CACTCCCAAAACCTGCTGAG | TCTCTTCAGAAGTGCAAGGGTA |
| IL12B | AGGGCAAGAGCAAGAGAGAA | CATTTTTGCGGCAGATGAC |
| IL4 | AGCTGATCCGATTCCTGAAA | GTTGGCTTCCTTCACAGGAC |
| IL13 | CAGTGCCATCGAGAAGACC | GGACATGCAAGCTGGAAAAC |
| GATA3 | CTCATTAAGCCCAAGCGAAG | TCTGACAGTTCGCACAGGAC |
| IL17A | CCCCAAAGCAGTTAGACTATGG | TTGAAGGATGAGGGTTCCTG |
| IL6 | AGAGGCACTGGCAGAAAACA | GCTCTGGCTTGTTCCTCACT |
| TNF | CAGCCTCTTCTCCTTCCTGAT | GCCAGAGGGCTGATTAGAGA |
| IL10 | AGGGAGCCCCTTGATGAT | GGTTGGGGAATGAGGTTAGG |
| TGFB | ACTACTACGCCAAGGAGGTCAC | TGCTTGAACTTGTCATAGATTTCG |
| CCL13 | TCTCTGCTTCTCATGCTGCT | TTGTGTAATAGACCTCCAGAACAC |
| CCL19 | GCTGGTTCTCTGGACTTCC | GGATGGGTTTCTGGGTCAC |
| CCL21 | GAACCAAGCTTAGGCTGCTC | CTTTGGGTCTGCACATAGCTC |
| CCR7 | CTCTTCAAGGACCTGGGCTG | GTCCCTCTAGTCCAGGCAGA |
| U6 | TCGGCAGCACATATACTAAAATTGG | ACGAATTTGCGTGTCATCCT |

Supplementary table 4. Univariate and multivariate analyses of prognostic factors associated with overall survival and recurrence-free survival in 240 HCC patients from West China Hospital.

| Variables | Overall survival | | | | Recurrence-free survival | | | |
| --- | --- | --- | --- | --- | --- | --- | --- | --- |
|  | Univariate analysis | | Multivariate analysis | | Univariate analysis | | Multivariate analysis | |
|  | HR (95%CI) | P value | HR (95%CI) | P value | HR (95%CI) | P value | HR (95%CI) | P value |
| Age (≥50/<50) | 0.751(0.539-1.047) | 0.091 |  |  | 0.803(0.563-1.146) | 0.227 |  |  |
| Gender (Female/Male) | 0.885(0.556-1.407) | 0.605 |  |  | 0.697(0.412-1.180) | 0.179 |  |  |
| HBsAg (+/-) | 2.328(1.223-4.431) | 0.010 | 2.388(1.236-4.614) | 0.010 | 1.899(0.994-3.628) | 0.052 |  |  |
| Cirrhosis (+/-) | 1.044(0.741-1.472) | 0.804 |  |  | 1.148(0.792-1.665) | 0.465 |  |  |
| Portal hypertension (+/-) | 0.883(0.544-1.435) | 0.616 |  |  | 1.176(0.728-1.900) | 0.506 |  |  |
| AFP (≥400/<400) | 1.214(0.868-1.697) | 0.257 |  |  | 1.734(1.215-2.474) | 0.002 | 1.420(0.972-2.076) | 0.070 |
| Tumor size (≥5/<5) | 1.705(1.204-2.414) | 0.003 | 1.203(0.823-1.757) | 0.340 | 1.741(1.201-2.526) | 0.003 | 1.341(0.909-1.979) | 0.139 |
| Tumor number (multiple/single) | 1.529(1.039-2.251) | 0.031 | 0.583(0.200-1.701) | 0.323 | 1.679(1.111-2.538) | 0.014 | 1.887(0.573-6.211) | 0.296 |
| Differentiation (Poor/Well-Moderate) | 1.131(0.804-1.590) | 0.479 |  |  | 1.162(0.809-1.670) | 0.416 |  |  |
| Macrovascular invasion | 4.516(2.393-8.522) | <0.001 | 1.785(0.704-4.523) | 0.222 | 3.829(1.975-7.423) | <0.001 | 1.848(0.695-4.916) | 0.219 |
| Microvascular invasion | 1.969(1.403-2.762) | <0.001 | 1.605(1.094-2.356) | 0.016 | 2.217(1.547-3.177) | <0.001 | 1.785(1.192-2.672) | 0.005 |
| BCLC (B-C/0-A) | 1.787(1.241-2.574) | 0.002 | 2.042(0.664-6.276) | 0.213 | 1.787(1.205-2.648) | 0.004 | 0.700(0.197-2.488) | 0.581 |
| TNM (Ⅲ/Ⅰ-Ⅱ) | 1.328(0.922-1.912) | 0.127 |  |  | 1.357(0.917-2.010) | 0.127 |  |  |
| pTLS (High/Low) | 0.418(0.298-0.585) | <0.001 | 0.437(0.308-0.618) | <0.001 | 0.437(0.306-0.625) | <0.001 | 0.507(0.326-0.789) | 0.003 |
| iTLS (+/-) | 0.745(0.513-1.081) | 0.121 |  |  | 0.571(0.375-0.871) | 0.009 | 0.893(0.537-1.487) | 0.665 |

AFP: alpha-fetoprotein; BCLC: Barcelona Clinic of Liver Cancer; TNM: tumor-nodes-metastasis; pTLS: peritumoral tertiary lymphoid structure; iTLS: intratumoral tertiary lymphoid structure; HR, hazard ratio; CI, confidence interval.

Supplementary table 5. Univariate and multivariate analyses of prognostic factors associated with overall survival and recurrence-free survival in 120 HCC patients from the Third Affiliated Hospital of Sun Yat-sen University.

| Variables | Overall survival | | | | Recurrence-free survival | | | |
| --- | --- | --- | --- | --- | --- | --- | --- | --- |
|  | Univariate analysis | | Multivariate analysis | | Univariate analysis | | Multivariate analysis | |
|  | HR (95%CI) | P value | HR (95%CI) | P value | HR (95%CI) | P value | HR (95%CI) | P value |
| Age (≥50/<50) | 0.779(0.466-1.300) | 0.339 |  |  | 1.250(0.747-2.092) | 0.396 |  |  |
| Gender (Female/Male) | 0.648(0.307-1.367) | 0.254 |  |  | 0.723(0.355-1.470) | 0.370 |  |  |
| HBsAg (+/-) | 1.040(0.494-2.194) | 0.917 |  |  | 1.168(0.530-2.572) | 0.700 |  |  |
| Cirrhosis (+/-) | 0.819(0.483-1.390) | 0.460 |  |  | 0.979(0.571-1.678) | 0.937 |  |  |
| Portal hypertension (+/-) | 0.846(0.383-1.870) | 0.680 |  |  | 1.396(0.707-2.756) | 0.337 |  |  |
| AFP (≥400/<400) | 0.980(0.574-1.676) | 0.942 |  |  | 1.742(1.041-2.915) | 0.034 | 1.312(0.760-2.264) | 0.329 |
| Tumor size (≥5/<5) | 1.106(1.029-1.189) | 0.006 | 1.053(0.976-1.137) | 0.181 | 1.063(0.982-1.150) | 0.128 |  |  |
| Tumor number (multiple/single) | 1.676(0.848-3.312) | 0.137 |  |  | 2.179(1.175-4.041) | 0.013 | 2.033(1.078-3.831) | 0.028 |
| Differentiation (Poor /Well-Moderate) | 0.953(0.565-1.607) | 0.857 |  |  | 1.151(0.688-1.925) | 0.592 |  |  |
| Macrovascular invasion | 0.772(0.107-5.587) | 0.798 |  |  | 3.447(1.554-7.646) | 0.002 | 2.629(1.128-6.124) | 0.025 |
| Microvascular invasion | 2.701(1.610-4.531) | <0.001 | 2.460(1.453-4.165) | 0.001 | 2.401(1.430-4.031) | 0.001 | 1.957(1.124-3.408) | 0.018 |
| BCLC (B-C/0-A) | 1.529(0.793-2.947) | 0.205 |  |  | 1.734(0.919-3.269) | 0.089 |  |  |
| TNM (Ⅲ/Ⅰ-Ⅱ) | 1.142(0.593-2.201) | 0.690 |  |  | 1.466(0.792-2.714) | 0.224 |  |  |
| pTLS (High/Low) | 0.445(0.264-0.749) | 0.002 | 0.457(0.271-0.771) | 0.003 | 0.394(0.236-0.658) | <0.001 | 0.389(0.229-0.660) | <0.001 |
| iTLS (+/-) | 0.633(0.352-1.138) | 0.126 |  |  | 0.606(0.337-1.090) | 0.095 |  |  |

AFP: alpha-fetoprotein; BCLC: Barcelona Clinic of Liver Cancer; TNM: tumor-nodes-metastasis; pTLS: peritumoral tertiary lymphoid structure; iTLS: intratumoral tertiary lymphoid structure; HR, hazard ratio; CI, confidence interval.

Supplementary table 6. Baseline characteristics of 120 HCC patients used for gene expression analyses.

| Variables | Entire cohort (n=120) | Low pTLS density (n=60) | High pTLS density (n=60) | P value |
| --- | --- | --- | --- | --- |
| Age (<50/≥50) | 66/54 | 37/23 | 29/31 | 0.199 |
| Gender (male/female) | 97/23 | 48/12 | 49/11 | 1.000 |
| HBsAg (+/-) | 105/15 | 52/8 | 53/7 | 1.000 |
| Cirrhosis (+/-) | 69/51 | 31/29 | 38/22 | 0.268 |
| Portal hypertension (+/-) | 18/102 | 10/50 | 8/52 | 0.799 |
| AFP, ng/dL (<400/≥400) | 63/57 | 28/32 | 35/25 | 0.378 |
| Tumor size, cm (<5/≥5) | 53/67 | 18/42 | 35/25 | 0.003 |
| Tumor number (single/ multiple) | 97/23 | 43/17 | 54/6 | 0.019 |
| Differentiation (well-moderate/poor) | 78/42 | 28/32 | 50/10 | 0.013 |
| Microvascular invasion (+/-) | 32/88 | 19/41 | 13/47 | 0.302 |
| BCLC stages (0-A/B-C) | 93/27 | 39/21 | 54/6 | 0.002 |
| TNM stages (Ⅰ-Ⅱ/Ⅲ) | 86/34 | 33/27 | 52/7 | 0.031 |
| iTLS (+/-) | 28/92 | 0/60 | 28/32 | <0.001 |

AFP: alpha-fetoprotein; BCLC: Barcelona Clinic Liver Cancer; TNM: tumor-nodes-metastasis; pTLS: peritumoral tertiary lymphoid structure; HCC: hepatocellular carcinoma.

Supplementary table 7. Comparing of baseline characteristics between frozen and FFPE HCC samples with low pTLS density in the training cohort.

| Variables | Frozen samples (n=60) | | FFPE samples (n=89) | P value |
| --- | --- | --- | --- | --- |
| Age (<50/≥50) | 37/23 | | 51/38 | 0.615 |
| Gender (male/female) | 48/12 | | 73/16 | 0.832 |
| HBsAg (+/-) | 52/8 | | 79/10 | 0.799 |
| Cirrhosis (+/-) | 31/29 | | 50/39 | 0.618 |
| Portal hypertension (+/-) | 10/50 | | 13/76 | 0.818 |
| AFP, ng/dL (<400/≥400) | 28/32 | | 42/47 | 0.422 |
| Tumor size, cm (<5/≥5) | 18/42 | | 28/61 | 0.859 |
| Tumor number (single/ multiple) | 43/17 | | 66/23 | 0.851 |
| Differentiation (well-moderate/poor) | 28/32 | | 58/31 | 0.145 |
| Microvascular invasion (+/-) | 19/41 | | 31/57 | 0.603 |
| BCLC stages (0-A/B-C) | 39/21 | | 62/27 | 0.594 |
| TNM stages (Ⅰ-Ⅱ/Ⅲ) | 33/27 | | 60/29 | 0.347 |
| iTLS (+/-) | | 0/60 | 0/89 | 1.000 |
| 5-year survival | 19 (31.7%) | | 28 (31.5%) | 1.000 |

AFP: alpha-fetoprotein; BCLC: Barcelona Clinic Liver Cancer; TNM: tumor-nodes-metastasis; pTLS: peritumoral tertiary lymphoid structure; HCC: hepatocellular carcinoma; FFPE, paraffin-embedded.

Supplementary table 8. Comparing of baseline characteristics between frozen and FFPE HCC samples with high pTLS density in the training cohort.

| Variables | Frozen samples (n=60) | FFPE samples (n=151) | P value |
| --- | --- | --- | --- |
| Age (<50/≥50) | 29/31 | 66/85 | 0.646 |
| Gender (male/female) | 49/11 | 129/22 | 0.531 |
| HBsAg (+/-) | 53/7 | 132/19 | 1.000 |
| Cirrhosis (+/-) | 38/22 | 100/51 | 0.749 |
| Portal hypertension (+/-) | 8/52 | 23/128 | 0.831 |
| AFP, ng/dL (<400/≥400) | 35/25 | 97/54 | 0.753 |
| Tumor size, cm (<5/≥5) | 35/25 | 76/75 | 0.359 |
| Tumor number (single/ multiple) | 54/6 | 127/24 | 0.288 |
| Differentiation (well-moderate/poor) | 50/10 | 90/51 | 0.165 |
| Microvascular invasion (+/-) | 13/47 | 49/102 | 0.134 |
| BCLC stages (0-A/B-C) | 54/6 | 123/28 | 0.150 |
| TNM stages (Ⅰ-Ⅱ/Ⅲ) | 52/7 | 118/33 | 0.534 |
| iTLS (+/-) | 28/32 | 76/75 | 0.650 |
| 5-year survival | 37 (61.7%) | 96 (63.6%) | 0.875 |

AFP: alpha-fetoprotein; BCLC: Barcelona Clinic Liver Cancer; TNM: tumor-nodes-metastasis; pTLS: peritumoral tertiary lymphoid structure; HCC: hepatocellular carcinoma; FFPE, paraffin-embedded.
